# Analysis of Heterozygous *BRCA1* 5382ins Founder Mutation in a Cohort of Egyptian Breast Cancer Female Patients Using Pyrosequencing Technique

**DOI:** 10.31557/APJCP.2020.21.2.431

**Published:** 2020

**Authors:** Salwa H Gomaa Mogahed, Yasser S Hamed, Yassmin E Ibrahim Moursy, Marwa H Mahomoud Saied

**Affiliations:** 1 *Department of Chemical Pathology, *; 2 *Department of Experimental and Clinical Surgery, Medical Research Institute, *; 3 *M.B.B.Ch, *; 4 *4Department of Chemical and Clinical Pathology, Faculty of Medicine, Alexandria University, Egypt. *

**Keywords:** Familial breast cancer, BRCA 1 mutation, Pyrosequencing technique

## Abstract

**Background:**

Up to half of the heritable mutations in breast cancer (BC) are attributed to *BRCA1* and *BRCA2 *genes. The mutation prevalence is variable based on ethnicity and may be influenced by founder mutations. The aim of this pilot study is to determine for the first time, the prevalence of *BRCA1* 5382insC founder mutation in a cohort of Egyptian familial breast cancer patients (FBC).

**Methods:**

Female patients were selected to have familial type of breast cancer. Twenty healthy females were included as a control group. Peripheral blood samples were withdrawn from all studied females and were analyzed for *BRCA1* 5382insC founder mutation detection using pyrosequencing technique.

**Results:**

Eighty Egyptian FBC females were eligible to be enrolled in the study with a mean age of 48.31 ± 10.97years.We found a *BRCA1* 5382insC mutation carrier frequency of 5% of total studied FBC patients (4 out of 80 patients) with 95% confidence interval (1.61-12.99). There was a high statistical significant difference between carriers and non-carriers concerning the number of affected family members by BC, (p=0.001).

**Conclusion:**

*BRCA1* 5382insC founder mutation is not uncommon among Egyptian FBC females. The carrier frequency is comparable to that reported worldwide; however it is lower than those from previous Egyptian studies using different molecular techniques. The strong association between the mutation and the number of affected family members suggest wider screening of the mutation among high risk families using the reliable pyrosequencing technique.

## Introduction

Breast cancer (BC) is the most frequently occurring cancer among women, representing a quarter of all diagnosed cancers (Bray et al., 2018). It is also the first cause of cancer-related deaths among them(DeSantis et al., 2015). In Egypt, according to the latest National Cancer Registry Program (NCRP), BC is the most common cancer among women representing 32.04% of total cancers (Ibrahim et al., 2014).

*BRCA1* and *BRCA2* (breast cancer predisposition gene 1/2)are the strongest susceptibility genes for BCaccounting for up to half of the heritable mutations in BC and inherited in an autosomal dominant pattern with incomplete penetrance (Tung et al., 2015).

Defective DNA double-strand repair is a characteristic of germline mutations in *BRCA1* and *BRCA2* genes in all cells expressing them (Mehrgou and Akouchekian, 2016).They are also classified as pathogenic variants or deleterious mutations that predispose to familial breast and or ovarian cancer (Felix et al., 2018; McCartan and Chatterjee, 2018).

These mutations vary among different populations as a result of founder effect (a mutation that occurs more frequently in a particular population) (Ossa and Torres, 2016). *BRCA1* 5382insC is one of the *BRCA1*founder mutations that was linked to Ashkenazi Jewish (Lieberman and Tomer, 2017) and has been implicated in hereditary and familial BC. It is a frame shift mutation in which there is an insertion of a cytosine nucleotide at the position of 5382 of exon 20 of *BRCA1* gene resulting into production of truncated premature nonfunctioning protein (Rebbeck et al., 2015).

According to Middle East Cancer Consortium(MECC), the young age at onset as well as high-grade tumor in Middle East and North Africa (MENA) suggest contribution of genetic factors such as *BRCA1*mutations (Chouchane et al., 2013; Laraqui et al., 2015). A large number of families and frequent consanguinity in the Arab population support the presence of founder mutations (Chouchane et al., 2013). Mutation Data from Egyptian families also suggest the presence of strong *BRCA1* and 2 founder effect in their population (Ibrahim et al., 2010).

Given the high penetrance rates among BRCA mutation carriers (depending on their frequency and level of risk), it will be certainly important to identify those who could benefit from the available preventive options such as bilateral prophylactic mastectomy in healthy carriers(Song et al., 2018) as well as those affected women who are indicated for the newly developed gene specific therapies in the era of personalized cancer treatment(Odle, 2017).

Although different molecular techniques have been described for BRCA mutation detection,DNA sequencing is definitely being considered the gold standard as it assisted in analysis of genes at single nucleotide level (Takano et al., 2008).

Pyrosequencing is a real-time sequencing method based on sequence by synthesis (SBS) principle with high throughput in the production of a short length of reads making it a reliable and excellent method for *BRCA1* founder mutation detection (Zhang et al., 2009).

Therefore, this study aimed at analysis of *BRCA1* 5382insC founder mutation in a cohort of Egyptian female population with familial BC and for the first time by pyrosequencing technique in order to obtain a reliable estimate for its frequency among them.

## Materials and Methods

Egyptian female patients diagnosed with familial breast cancer (FBC), with a family history of BC in one or more of their first-degree relatives were consecutively enrolled in the study. They were recruited from the Clinical Surgery or medical oncology clinics of Medical Research Institute (MRI), Alexandria University. Twenty age matched healthy females, visiting the breast clinic for routine mammography and with negative family history of BC, were also included as a control group. Written informed consents were taken from all studied subjects. The study was approved by the Ethical Committee of the MRI, Alexandria University. Data were collected from patients’ medical records including histopathology of the tumor, cancer stage, presence of metastasis, mammography and fine needle aspiration cytology results as well as hormone receptors and HER2 status. Finally, detection of *BRCA1* 5382insC mutation in peripheral blood samples from all studied females was done using a pyrosequencing technique.

*Pyrosequencing steps*

*DNA extraction*

DNA was extracted from peripheral blood specimens with Thermo Scientific Gene JET Whole Blood Genomic DNA Purification Mini Kit (Catalog No. K0781) following the manufacturer’s protocol. Then the concentration and quality of the purified genomic DNA were assessed by thermoscientificNanoDropTM1000 spectrophotometer.

*PCR amplification and visualization of the target DNA*

**Figure 1:**
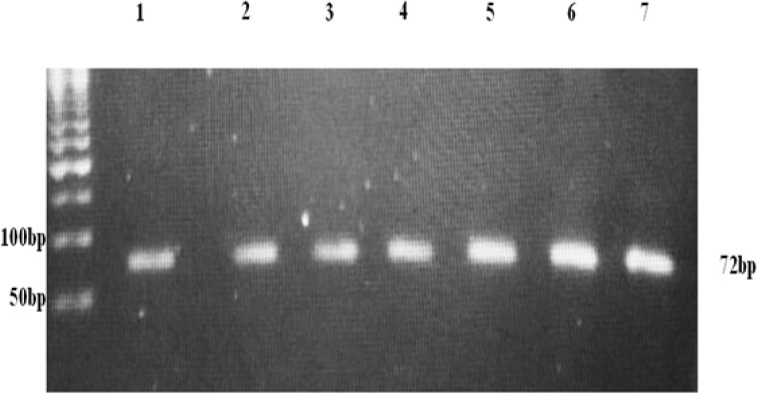
Visualization of PCR Products on Gel Prior to Pyrosequencing. 50 bp Ladder (on the Left) and the Product Band at 72 bp for Seven Different Samples (on the Right).

A PyroMark PCR Kit from QIAGEN (Catalog No. 978703) was selected since it enables highly specific and unbiased amplification of template DNA. For each PCR reaction the following were added: 12.5 μl of PyroMark PCR MasterMix, 3 μl Primer (100 μmol), 2.5μlCoralLoadConcentrate, 4.5 μl RNase-Free Water and 2.5 μl Template DNA to reach a final volume of 25μl.We used two oligonucleotide primers (forward and reverse) (Zhang et al., 2009) that flank the mutation locus:*BRCA1* 5382insC forward: 5′-AAAGCGAGCAAGAGAATCCC-3′and *BRCA1* 5382insC reverse:5′-TGGGGTGAGATTTTTGTCAAC-3′-biotin.(One of the primers must be biotin-labeled to enable binding of the PCR product to streptavidin-coated beads during the preparation of single-stranded Pyrosequencing template), Table (1). PCR was performed using PCR Machine (Arktik Thermal Cycler, Thermo Scientific, USA).The PCR conditions were optimized and programmed as shown in Table 2. Then, all PCR amplicons were checked over 2% agarose gel electrophoresis to ensure the presence of a single clear band (without secondary product or primer dimer) before pyrosequencing, Figure 1.

*Pyrosequencing detection of heterozygous BRCA1 5382insC mutation*

**Figure 2:**
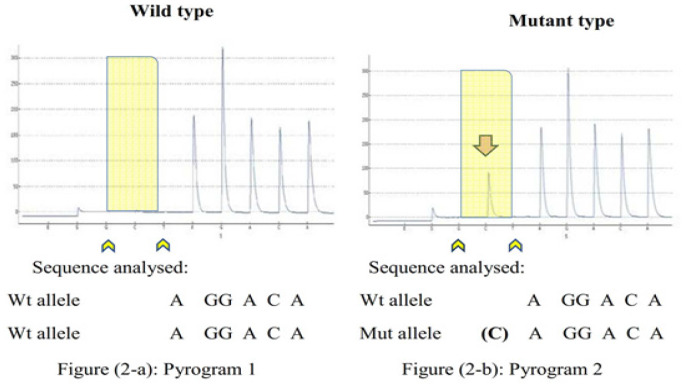
Pyrograms Showing Heterozygous Insertion of C Indicated by an Arrow that is Present in the Mutant (2-b), but not in the Wild-Type (2-a) of *BRCA1* 5382insC. Pyrograms at positions 1 and 3 shows one unit G and one unit T (arrowheads) which are negative nucleotide dispensations that serve as internal controls for nucleotide misincorporation

Using QiagenPyroMark Q24 Gold kit (Catalog No. 970802), the PCR products were analyzed for *BRCA1* 5382insC mutation by sequencing on a PyroMark Q24 following the manufacturer’s instructions using the pyrosequencing primer: 5′-CGAGCAAGAGAATCCC-3′, Table 1. Sequences to be analyzed and nucleotide dispensation order are shown in Figure 2 under each pyrogram.

*Design of the pyrosequencing assay*

The assay was designed to start sequence analysis right at the mutation site (*BRCA1* 5382insC). Then, the PyroMark Q24 was programmed with the protocol of sequential nucleotide dispensation. Besides, negative nucleotide dispensations were inserted to serve as internal controls to avoid nucleotide misincorporation. Peak heights are proportional to the nucleotidesˈ numbers that are incorporated with each dispensation. 

*Analyzing the data by PyroMark Q24 Software and obtaining the results*

PyroMark Q24 Software, installed on a personal computer, enables analysis of the results in the form of pyrogram for each sample.

*Statistical Analysis*

Statistical analysis was done using SPSS program version 20 (Statistical Package of Social Sciences, Chicago, USA). The distributions of quantitative variables were tested for normality using Kolmogorov-Smirnov test. Normally distributed test results were represented in the form of Mean ± Standard deviation. Independent samples t-test was used to compare quantitative variables between 2 groups. The data of the nominal variables were summarized in the form of frequencies and percentages. The Chi-Square test (χ^2^ test) was used to compare proportions of *BRCA1*carriers and non-carriers according to nominal clinical data variables. When more than 20% of the cells have expected count less than 5, correction for chi-square was conducted using Fisher’s Exact test or Monte Carlo correction. Significance of the obtained results was judged by p- value ˂0.05 (Daly and Bourke, 2008).

## Results

Between 2017 and 2018, a total of 80 eligible FBC females were included in the study together with the 20 healthy volunteers. All demographic and clinical data as regards age of presentation, all reproductive factors as well as tumor stage and immunohistochemistry examination of the breast tumor tissues (hormone receptor profile and molecular subtypes of BC) are shown in Table 3.

*Interpretation of the pyrograms*

Following pyrosequencing analysis of samples, pyrograms were extracted and interpreted. As shown in Figure 2, incorporation of an extra nucleotide C, in case of *BRCA1* 5382insC mutation, was seen in the mutant allele, Figure (2-b) which is absent in the wild type, Figure 2-a, (a very clear distinct pyrogram). As the peak intensity of the C (one light unit) is approximately half of other peaks at positions 4, 6, 7, 8, it represents insertion of C in only one of the alleles (heterozygous insC). All mutant genotypes were confirmed by repeat analysis.

*BRCA1 5382insC mutation carriers and non-carriers*

As regards *BRCA1* 5382insC heterozygous mutation, the present study found a carrier frequency of 5% of total studied FBC Egyptian patients (4 out of 80 patients) with 95% confidence interval (1.61-12.99). Most of patients had (Luminal A) molecular subtype as (63.2% among non-carriers) and (75% among carriers). Detailed description and clinical characteristics of each carrier are shown in Table (4). Notably, none of the healthy females (0/20, 0%) had the *BRCA1* 5382insC heterozygous mutation.

*Association of BRCA mutation carrier status and the clinical parameters*

Therefore, according to the carrier status, the studied FBC patients were further divided into two subgroups (carriers and non-carriers) to investigate possible associations of *BRCA1* 5382insC mutation with clinical representations of patients, Table 5.

**Table 1:** The PCR Primers and Sequencing Primer Used for *BRCA1* 5382insc Founder Mutation Analysis by Pyrosequencing

Mutation	Primer name	Primer sequence*	Product size(bp)
	5382insC-F	5′-AAAGCGAGCAAGAGAATCCC-3′	
BRCA1 5382insC	5382insC (biotinylated)	5′-TGGGGTGAGATTTTTGTCAAC-3′-biotin	72
	Sequencing primer	5′-CGAGCAAGAGAATCCC-3′	

*The GenBank references for primer sequences are: BRCA1 (NM 007304)

**Table 2:** PCR Cycling Protocol

Optimized cycling protocol	
Initial PCR activation step	15 min at 94°C
3-step cycling: Denaturation	30 sec at 94°C
Annealing	30 sec at 55°C
Extension	30 sec at 72°C
Number of cycles	45 cycle
Final extension	10 min at 72°C

**Table 3:** Distribution of the Studied Familial Breast Cancer Patients According to Demographic and Clinical Parameters (N = 80)

	No.	%
Age of first presentation (years)	48.31 ± 10.97	
≤50	44	55.00
>50	36	45.00
No. of affected family members		
One relative	57	71.25
Two relatives	14	17.50
Three relatives	9	11.25
Different types of cancers in the relatives of FBC patients		
Breast	101	90.18
Others	11	9.82
Menstrual history		
Premenopausal	46	57.50
Menopausal	34	42.50
Marital status		
Not married	10	12.50
Married	70	87.50
Parity		
Nulliparous	11	13.75
Multiparous	69	86.25
Breast feeding	69	86.25
Clinical presentation		
Breast lump	70	86.42
Others	11	13.58
Mammography		
BIRADS IV	35	43.75
BIRADS V	35	43.75
Others	10	12.50
Histopathological type		
IDC	67	83.75
Others(ILC,DCIS and mixed mucinous)	13	16.25
TNM Stage		
II	37	46.25
III	22	27.50
IV	11	13.75
Others (stage 0 and I)	10	12.50
Non-metastasis	69	86.25
Metastasis	11	13.75
Hormone receptor and HER2 status		
ER+ PR+ HER2-ve (Luminal A)	51	63.75
ER+ PR+ HER2+ve (Triple positive)	10	12.50
HER2+ve (HER2 enriched)	10	12.50
ER- PR- HER2-ve (Triple negative)	9	11.25
Carrier status of BRCA1 5382insC mutation		
Non-carrier	76	95
Carrier	4	5

IDC, Infiltrating ductal carcinoma; ILC, Invasive lobular carcinoma; DCIS, Ductal carcinoma in situ

**Table 4.** Clinical Description of *BRCA1* 5382insC Mutation Carriers

	Carrier 1	Carrier 2	Carrier 3	Carrier 4
Age of first presentation (years)	40	32	48	41
Menopausal state	Premenopause	Premenopause	Premenopause	Premenopause
	Sister BC	Sister BC died	MotherBC died	Mother BC
Family history	Father brain	Aunt BC died	Aunt bilateral BC	Aunt BC
	Brother lung	Uncle GIT		Sister BC
Mammography	BIRADS IV	BIRADS V	BIRADS IV	BIRADS V
Pathology	IDC	IDC	IDC	IDC
Cancer stage	III	III	II	IV
Visceral metastasis	Free	Free	Free	Free
Bone metastasis	Free	Free	Free	Positive
Hormone receptors and HER2 status	ER, PR -ve	ER, PR +ve	ER, PR +ve	ER, PR +ve
	HER2 +ve	HER2 -ve	HER2 -ve	HER2 -ve
	HER2 enriched	Luminal A	Luminal A	Luminal A
Survival	Still alive	Still alive	Still alive	Still alive

BIRADS, Breast Imaging; Reporting and Data System IDC, Infiltrating ductal carcinoma; ILC, Invasive lobular carcinoma; DCIS, Ductal carcinoma in situ; ER, Estrogen receptors; PR, Progesterone receptors; HER2, The human epidermal growth factor receptor 2; BRCA1, Breast cancer gene 1

**Table 5:** Comparison between Mutation Carrier and Non-Carrier Cases according to Their Clinical Parameters

		Non-carrier				Carrier				FEp
		(n = 76)				(n = 4)				
		No.		%		No.		%		
Age of first presentation (years)		48.74 ± 11.01				40.25 ± 6.55				0.132
≤50		40		52.6		4		100		0.123
>50		36		47.4		0		0		
Menstrual history										
Premenopausal		42		55.3		4		100		0.133
Menopausal		34		44.7		0		0		
Marital status										
Not married		8		10.5		2		50		0.074
Married		68		89.5		2		50		
Parity										
Nulliparous		9		11.8		2		50		0.089
Multiparous		67		88.2		2		50		
Breast feeding										
Negative		9		11.8		2		50		0.089
Positive		67		88.2		2		50		
Mammography										
BIRADS I		1		1.32		0		0		1
BIRADS II		1		1.32		0		0		
BIRADS III		6		7.89		0		0		
BIRADS IV		33		43.42		2		50		
BIRADS V		33		43.42		2		50		
BIRADS VI		2		2.63		0		0		
Histopathological type										
IDC		63		82.9		4		100		1
ILC		9		11.84		0		0		
DCIS		2		2.63		0		0		
Mixed mucinous carcinoma		2		2.63		0		0		
	Non-carrier				Carrier				FE_p_	
	(n = 76)				(n = 4)					
	No.		%		No.		%			
TNM Stage										
0	2		2.6		0		0		0.619	
I	8		10.5		0		0			
II	36		47.4		1		25			
III	20		26.3		2		50			
IV	10		13.2		1		25			
Metastasis										
Negative	66		86.8		3		75		0.453	
Positive	10		13.2		1		25			
ER+ PR+ HER2- ve(Luminal A)	48		63.2		3		75		1	
ER+ PR+ HER2+ve(Triple +ve )	10		13.2		0		0		1	
ER-PR-HER2+ve (HER2 enriched)	9		11.8		1		25		0.42	
ER- PR- HER2- ve(Triple negative)	9		11.8		0		0		1	
Family history										
One relative	57		75.0		0		0		0.001*	
Two relatives	13		17.1		1		25			
Three relatives	6		7.9		3		75			
1 year Survivability										
Unknown	3		3.9		0		0		1	
Died	5		6.6		0		0			
Survived	68		89.5		4		100			

p, p-value for comparing between the two groups; *, Statistically significant at p ≤ 0.05; FE, Fisher exact test

## Discussion

Data related to familial and hereditary BC among the Arab population are rare with few reported from Egypt, however they support the presence of BRCA founder mutation in this population.(Ibrahim et al., 2010; Chouchane et al., 2013). 

*BRCA1* 5382insC, is the second most recurrent mutation reported in the *BRCA1* gene in different countries, according to the breast cancer information core (BIC) (Odle, 2017).

The present study reported for the first time the prevalence of *BRCA1* 5382insC founder mutation among Egyptian FBC patients using pyrosequencing technique. The heterozygous mutation was detected in 4 out of 80 patients with a carrier frequency of 5% (95% confidence interval 1.61-12.99), Table 3. Worldwide population studies have revealed comparable mutation frequency in different countries; as in Ashkenazi Jewish (6% early onset BC), Dagan et al., (2017) in Greek (5.5% in breast /ovarian cancer families), Konstantopoulou et al., (2014) in South Africa (3.3% in breast and /or ovarian cancer families) Reeves et al., (2004) and other populations worldwide (Backe et al., 1999; Jasinska and Krzyzosiak, 2001; Sokolenko et al., 2006; Fernandes et al., 2016).

To date, no published data from Egypt about *BRCA1* 5382insC prevalence using pyrosequencing. After serious search, we found only one Egyptian study that determined another *BRCA1* (185delAG) founder mutation using pyrosequencing technique and reported a carrier frequency of 2.5% among the studied BC females (Saied et al., 2017).

Some studies from Egypt discussed *BRCA1* and or *BRCA2* mutations using other techniques. For example, a previous work by (Abdel-Mohsen et al., 2016) revealed the presence of *BRCA1* 5382insC in 66.7% of BC patients compared with 10% among healthy controls using methylation specific-PCR and PCR-RFLP.

On the contrary, a very recent study by (Abou-El-Naga et al., 2018) found a higher frequency of *BRCA1* 5382insC among BC patients (11.6%) relative to *BRCA1* 185delAg (2.3%) but they also found a higher frequency of *BRCA1* 5382insC mutation among unrelated controls (49.5%) and first-degree relatives of mutation carriers (6.3%), using multiplex-PCR technique. 

The variability of our results from those of other studies may be attributed to difference in methodology of testing, criteria used for patient selection and number of studied populations as well. As regards the methodology, those based on digestion approach are suspected to have lower performance as the other mutations that could be present in the targeted *BRCA1* gene isolates could alter the restriction enzyme recognition sites causing false-negative or false-positive results. In addition, compared to Sanger sequencing, pyrosequencing is cost effective and ideal for short fragments sequencing (Fuller et al., 2016). 

In the current study, the mean age of studied FBC patients was 48.31 years old, Table 3. The mean age of first presentation in *BRCA1* 5382insC mutation carriers (40.25 years) was lower than that of non-carriers (48.74 years) with no statistically significant difference, Table (5).This was in concordance with a very recent research by Kwong et al., (2018) who found younger age at presentation (42.36 years) in BRCA mutations than that of the studied high risk group (47.11 years). Similar finding was reported by Ibrahim et al., (2010) and Cronin-Fenton et al., (2017).

The present study showed that BC was the most prevalent cancer among relatives of carriers and non-carriers as it represents 90.18% of all cancers among them, Table 3. *BRCA1* 5382insC carriers had relatives with BC, bilateral BC, GIT, lung and brain tumors, Table 4. Ozsoy et al., (2017) suggested that family history of BC is the most important risk factor among all other risk factors. Similarly, other studies such as(Jasinska and Krzyzosiak, 2001; Cherbal et al., 2010) found a strong family history of BC in all carriers of *BRCA1* 5382insC mutation. 

In the present study 3 out of 4 (75%) of the carrier group had 3 family members affected with cancers, Table 5. In line with our study, Pajares et al., (2018) reported that among the studied *BRCA1*-mutated families, the most frequent criterion was the presence of three or more family members with breast and/or ovarian cancer.

Interestingly, the present study revealed for *BRCA1* 5382insC carrier number (1), that the affected first degree relatives were from the father side, since father, brother and sister of the same patient had cancers (brain, lung and breast cancers respectively), Table 4. Aggregation of such tumors with BC in the same family draw the attention to family cancer syndrome with various cancers among family which most probably due to a tumor suppressor gene mutation. Therefore, for all carriers identified, a complete screening for coding regions of BRCA genes is necessary. Notably, it had been reported that TP53 mutations were found in concomitant with *BRCA1* associated breast tumors Crook et al., (1997). Consequently, targeted deep sequencing of common variants in both BRCA and TP53 genes in those carriers is essential. 

Concerning the hormone receptor and HER2 status (ER, PR, and HER2), our study showed no statistical significant difference between carrier and non-carrier groups. Only an increased incidence of ER+ PR+ BC among studied FBC patients was identified, Table 5. Molino et al., (2004) reported that BC patients with a positive family history were more likely to have ER+ tumors with no significant association between PR+ tumors and family history. Remarkably, in the present study, triple negative BC was found in only 11.25% of FBC patients (9/80), Table 3 and none was a carrier for *BRCA1* 5382insC mutation, Table 5.

On contrary, other studies found that triple negative BC was more common in BRCA mutations carriers, while triple positive tumors were more common in non-carriers. (Alemar et al., 2017; Chen et al., 2018). Furthermore, HER2-positive phenotype is a molecular subtype not frequently associated with BRCA deficiency (Maynes et al., 2010). Interestingly, carrier number (1) in our study had HER2 enriched phenotype, Table (4), and showed a good response to targeted chemotherapy i.e. Herceptin.

Moreover, in the current study, there were no statistical significance differences as regards Tumor stage, distant metastasis and one year survivability between carrier and non-carrier groups, Table 5. 

The association between BRCA mutations and survivability is controversial as BC prognosis in BRCA mutation carriers remains poorly understood. In line with current study, Yadav et al., (2018) found no statistical significant difference in overall survival (OS) between the BRCA mutation carriers and non-carriers. Conversly, (van den Broek et al., 2015) were found to be heterogenous and indecisive. Also, Schmidt et al., (2017) concluded a worse prognosis among *BRCA1* or *BRCA2* mutation carriers diagnosed with BC before age of 50 years. They explained their findngs by difference in tumor characteristics, treatment response and second ovarian cancers.

We conclude from our study that *BRCA1* 5382insC founder mutation was detected in the studied Egyptian familial breast cancer (FBC) female patients with 5% carrier frequency that was comparable to worldwide frequencies, but lower than those reported from earlier Egyptian studies. *BRCA1* 5382insC mutation carriers are younger than non-carriers at their first presentation. Moreover, a strong association was found between occurrence of *BRCA1* 5382insC mutation and the number of affected family members by BC. Therefore, we suggest wider screening of the mutation among high risk families in Egypt using pyrosequencing technique that could be an excellent platform for BRCA founder mutation analysis.
